# Multimodal MRI radiomic models to predict genomic mutations in diffuse intrinsic pontine glioma with missing imaging modalities

**DOI:** 10.3389/fmed.2023.1071447

**Published:** 2023-02-23

**Authors:** Fahad Khalid, Jessica Goya-Outi, Thibault Escobar, Volodia Dangouloff-Ros, Antoine Grigis, Cathy Philippe, Nathalie Boddaert, Jacques Grill, Vincent Frouin, Frédérique Frouin

**Affiliations:** ^1^Laboratoire d'Imagerie Translationnelle en Oncologie (LITO)-U1288, Institut Curie, Inserm, Université Paris-Saclay, Orsay, France; ^2^DOSIsoft SA, Cachan, France; ^3^Department of Paediatric Radiology, Hôpital Universitaire Necker Enfants Malades, Paris, France; ^4^Institut Imagine, Inserm U1163 and U1299, Université Paris Cité, Paris, France; ^5^Neurospin, Institut Joliot, CEA, Gif-sur-Yvette, France; ^6^Département Cancérologie de l'enfant et de l'adolescent, Gustave-Roussy, Villejuif, France; ^7^Prédicteurs moléculaires et nouvelles cibles en oncologie-U981, Inserm, Université Paris-Saclay, Villejuif, France

**Keywords:** MRI, radiomics, prediction, missing data, genomic mutation, diffuse intrinsic pontine glioma

## Abstract

**Purpose:**

Predicting H3.1, TP53, and ACVR1 mutations in DIPG could aid in the selection of therapeutic options. The contribution of clinical data and multi-modal MRI were studied for these three predictive tasks. To keep the maximum number of subjects, which is essential for a rare disease, missing data were considered. A multi-modal model was proposed, collecting all available data for each patient, without performing any imputation.

**Methods:**

A retrospective cohort of 80 patients with confirmed DIPG and at least one of the four MR modalities (T1w, T1c, T2w, and FLAIR), acquired with two different MR scanners was built. A pipeline including standardization of MR data and extraction of radiomic features within the tumor was applied. The values of radiomic features between the two MR scanners were realigned using the ComBat method. For each prediction task, the most robust features were selected based on a recursive feature elimination with cross-validation. Five different models, one based on clinical data and one per MR modality, were developed using logistic regression classifiers. The prediction of the multi-modal model was defined as the average of all possible prediction results among five for each patient. The performances of the models were compared using a leave-one-out approach.

**Results:**

The percentage of missing modalities ranged from 6 to 11% across modalities and tasks. The performance of each individual model was dependent on each specific task, with an AUC of the ROC curve ranging from 0.63 to 0.80. The multi-modal model outperformed the clinical model for each prediction tasks, thus demonstrating the added value of MRI. Furthermore, regardless of performance criteria, the multi-modal model came in the first place or second place (very close to first). In the leave-one-out approach, the prediction of H3.1 (resp. ACVR1 and TP53) mutations achieved a balanced accuracy of 87.8% (resp. 82.1 and 78.3%).

**Conclusion:**

Compared with a single modality approach, the multi-modal model combining multiple MRI modalities and clinical features was the most powerful to predict H3.1, ACVR1, and TP53 mutations and provided prediction, even in the case of missing modality. It could be proposed in the absence of a conclusive biopsy.

## 1. Introduction

The diffuse intrinsic pontine glioma (DIPG) is a highly aggressive pediatric tumor, with a median overall survival of 11 months (1, 2). Since this tumor is inoperable, radiotherapy is the standard option that can be proposed systematically, generating in most cases transient improvement (3). Genomic analyzes based on tumor biopsies have shown that more than 85% of patients with DIPG harbor mutations (4, 5) at genes encoding histone H3, leading to lysine 27 to methionine substitution (H3-K27M). The new WHO classification of this disease is diffuse midline gliomas, H3 K27-altered (6). Most frequent H3-K27 alterations are H3.1 and H3.3 variants. These two alterations and the H3-wildtype are associated with different age profiles and different overall survivals, patients with H3.1 being younger, having better response to radiotherapy and better overall survivals (1). Furthermore, these H3 K27M mutations are frequently associated with TP53 and ACVR1 somatic mutations (7). If TP53 mutations are mainly encountered in H3.3 patients while ACVR1 mutation mostly occur in H3.1 patients, these mutations need to be separately identified for testing new chemotherapy options. It was recently shown that TP53 mutation can drive radio-resistance in patients with DIPG (8). Thus, the knowledge of this mutation could help to refine re-irradiation strategies. Furthermore, the combination of vandetanib and everolimus was identified as a possible therapeutic option for patient harboring ACVR1 mutations (9). These recent advances in the DIPG patient care, raised the issue of predicting H3.1, ACVR1, and TP53 mutations within tumor independently from each other, using data available at diagnosis time: basic clinical data (age and sex) and multi-modal MRI to help define a personalized treatment strategy when brain biopsy is not possible or is not conclusive.

Indeed, multi-modal MRI images are always acquired to confirm diagnosis (10, 11). These data could also be used for radiogenomic prediction tasks, provided that some pre-processing steps are taken. Radiomics is a recent field of research which refers to the comprehensive and automated quantification of this radiographic phenotype (12, 13). This approach aims at enhancing some relevant information contained in the images and made them available to clinicians. It is based on medical image post-processing algorithms and features computation from specific regions of interest (14–16). Radiomic features belong to different families, including morphological, global image intensity, histogram image intensity distribution and texture families. Texture indices are based on image intensity comparison between neighboring voxels, and potentially reflect biological properties such as tumor heterogeneities (12, 17, 18). The high number of radiomic features and their systematic analysis have accelerated the discovery of potential new biomarkers and has definitively modified the research tools in radiology and nuclear medicine, giving a larger weight to data analysis. However, end-users of radiomic tools should be aware of the pitfalls inherent in these tools (16, 19), including the dependency of radiomic features values to the acquisition parameters and to software implementation, and thus the need of image preprocessing to make these features more reproducible.

Magnetic resonance imaging (MRI) with its high spatial resolution and high brain tissue contrast is the imaging modality of choice for children with central nervous system tumors. Current recommendations include the acquisition of T1-weighted images without contrast (T1w) and following the injection of gadoterate meglumine (T1c), T2-weighted images (T2w), and fluid attenuated inversion recovery images (FLAIR) (10, 11). As MRI intensities are non-standardized (20), this prevents the extraction of robust radiomic features, except if specific standardization procedures are defined, including the use of similar pulse sequence parameters and identical size of voxels, and applying image intensity normalization as a preprocessing step (21–23). Of course, intensity variations depend on the MR scanner and the acquisition parameters, but also on each acquisition. To reduce this variability, many approaches have been proposed (24), including Z-score normalization, and dedicated procedures using a reference tissue, such as white matter for brain studies (25). A refined procedure was proposed for patients with DIPG, removing the slices corresponding to pontine location to avoid the inclusion of the tumor in the normalization process (21). However, despite intensity standardization, some variations in the radiomic features can be due to coils, scanners and/or scanning parameters as it was demonstrated on a breast phantom study (26). To reduce this impact, the ComBat method, providing harmonization of radiomic features across different acquisition scanners (27, 28), has been proposed.

In the constitution of our global approach, two specific issues were taken into account: 1) missing data: due to practical constraints some MRI modalities were missing or non-usable; 2) data scarcity for the training of our model: the cohort of patients was small, since DIPG is a rare disease. A compromise was made to incorporate as much relevant information as possible. In a preliminary work of our group, a radiomic model was proposed to distinguish the two types of histone H3-K27M mutations (H3.3 vs. H3.1) using a subset of patients having the four MRI modalities (T1w, T1c, T2w, and FLAIR) and clinical data (29). To increase the number of patients (about 20% for each prediction task), all the patients having at least one of the four MRI modalities were included. To have a prediction for each patient, a multi-model strategy was proposed using all the data types among clinical, T1w, T1c, T2w, and FLAIR that were available.

## 2. Materials and methods

### 2.1. Patient database

This retrospective mono-centric study includes 80 patients having DIPG, who had biopsy and were treated between 2009 and 2018 at Gustave Roussy cancer center (Villejuif, France). Patients were scanned at the time of diagnosis, before biopsy, with either Signa HDx, 1.5T (GE Healthcare) MRI machine or Discovery MR750w, 3T (GE Healthcare) MRI scanner in the pediatric radiology department at Necker Hospital (Paris, France).

**Figure 1:**
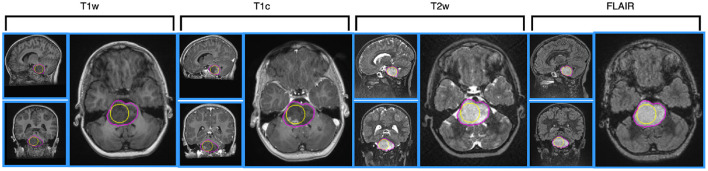
Illustration of MRI data for a 4 year-old patient, having H3.1 and ACVR1 mutations, having no TP53 mutation. MRI data are shown after intensity normalization using the hybrid white stripe method. From the left side to the right side: T1w, T1c, T2w and FLAIR images, using $\frac{\text{Sagittal}}{\text{Coronal}}|$ Axial layout for each modality. The contours of the sphere used for computing intensity and texture radiomic features inside the tumor are outlined in yellow color on each view. The contours of the tumor used for computing shape features are outlined in pink color.

**Figure 2:**
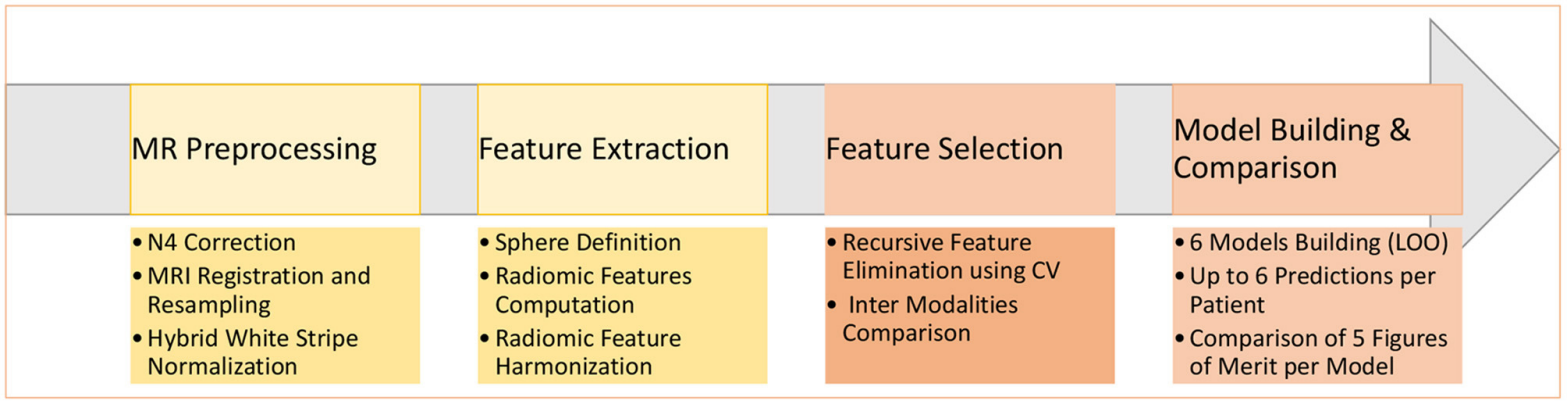
Main steps of the construction of the six machine learning models to predict a molecular mutation.

At least one of the four structural MRI modalities (T1w, T1c, T2w, and FLAIR) (see Table 1) was acquired and basic clinical information (age and sex) was also collected. Typical acquisition parameters were described in Goya-Outi et al. (21). Figure 1 shows a patient case from the database. A total of 57 (71%) patients had the four MRI modalities (T1w, T1c, T2w, and FLAIR) of sufficient quality, the remaining patients had at least one missing MRI modality. Following the genomic analysis consecutive to biopsy, 63 patients have known H3 status, 63 patients (partly different from the H3 subgroup) have known ACVR1 status and 61 patients have known TP53 status, as summarized in Table 2. For histone H3, the H3.1 mutation was observed in 14 patients, the H3.3 mutation in 44 patients, the H3.2 mutation in 1 patient and histone H3 wild type in 4 patients. Due to the small numbers in the last two classes, the binary task was to predict patients with H3.1 mutation against all other patients grouped together. Three binary tasks of classification were thus defined: prediction of H3.1, ACVR1, and TP53 mutations. Figure 2 gives an overview of the construction of the model which is defined for each prediction task, and the different steps are detailed in the following subsections.

**Table 1:** Number of modalities available according to each type of MR scanner.

**MR scanner type**	**T1w**	**T1c**	**T2w**	**FLAIR**
1.5 T	60	58	60	56
3 T	13	14	14	11

**Table 2:** Main clinical (age and sex) and molecular features.

	**Number of patients (F/M)**	**Mean Age (y)**
All patients	80 (35/45)	8.1 ± 4.4
**Histone H3 mutation status**		
Known	63 (30/33)	7.7 ± 3.7
H3.1	14 (8/6)	5.0 ± 1.6
H3.2	1 (0/1)	4.5
H3.3	44 (20/24)	8.7 ± 3.7
WT	4 (2/2)	6.9 ± 4.6
Others	49 (22/27)	8.5 ± 3.8
Unknown	17 (5/12)	9.3 ± 6.4
**ACVR1 mutation status**		
Known	63 (28/35)	7.9 ± 3.6
ACVR1 mutation	14 (7/7)	5.9 ± 3.0
WT	49 (21/28)	8.4 ± 3.6
Unknown	17 (7/10)	8.8 ± 6.6
**TP53 mutation status**		
Known	61 (29/32)	7.8 ± 3.7
TP53 mutation	34 (14/20)	9.0 ± 3.4
WT	27 (15/12)	6.3 ± 3.5
Unknown	19 (6/13)	8.8 ± 6.2

For Histone H3, “Others” gather H3.2 mutation, H3.3 mutation and Wild-Type (WT).

### 2.2. MRI preprocessing and radiomic features extraction

All MR Images were first processed through a dedicated pipeline fully described in Goya-Outi et al. (21) including bias field correction of MRI using N4 algorithm (30), MRI intensity normalization according to an adaptation of the hybrid white stripe approach (25), resampling to isotropic voxels of 1 mm^3^ and multimodal image registration on each T2w scan (when available, T1w or FLAIR otherwise) using FSL FLIRT (31).

For each patient, a spherical region was drawn (the largest sphere within the tumor) and transferred to the realigned MR volumes. This region always included the location of the biopsy. For each MRI modality, 79 radiomic features were extracted within the spherical region using PyRadiomics (14), including 19 first-order features derived from the distribution of intensity inside the tumor and 60 texture features computed using three different matrices: the gray level co-occurrence matrix (GLCM), the gray level run length matrix (GLRLM), and the gray level size zone matrix (GLSZM). All histogram-based and texture-based features were computed with a fixed bin width equal to 2 (21). As the MRI were acquired using two different scanners, the ComBat harmonization (27, 28) was then applied independently to each radiomic feature to make it more comparable across scanners (32). The spherical region is quick and easy to define and has already shown some promising results (32), but it does not bring any information related to the shape of the tumor. To overcome this drawback, tumor contours were delineated by two skilled operators and 14 additional shape features were extracted. As these features were available for each patient, they were further merged with clinical data. Results of this additional study are provided in Supplemental data.

### 2.3. Feature selection

A recursive feature elimination cross-validation (RFE-CV) method (33) was used to select the most relevant features. This procedure was repeated for each of the three classification tasks and for each modality *m* (1 ≤ *m* ≤ 5). It was implemented using the scikit-learn, a free machine learning library in Python (34). The RFE-CV method iteratively fits a model—a logistic regression model was chosen for our application—and removes progressively the weakest feature. Therefore, the RFE-CV method eliminates dependencies and co-linearity between the different features in the model. To apply the L1 penalty used for the logistic regression model, we used a grid analysis introducing a variation (between 0.1 and 1 with a step size of 0.1) for the inverse of the regularization strength, the *C* parameter. Feature importance was assessed on the validation set by computing the Brier score loss. The RFE process was repeated 40 times, based on a two-fold cross-validation. The up to four most frequently selected features were kept. The RFE-CV provided a subset of *K* features $f_{m}^{k}$, 1 ≤ *k* ≤ *K*, associated with the modality *m*.

### 2.4. Definition of the mono-modal and multi-modal models

**Figure 3:**
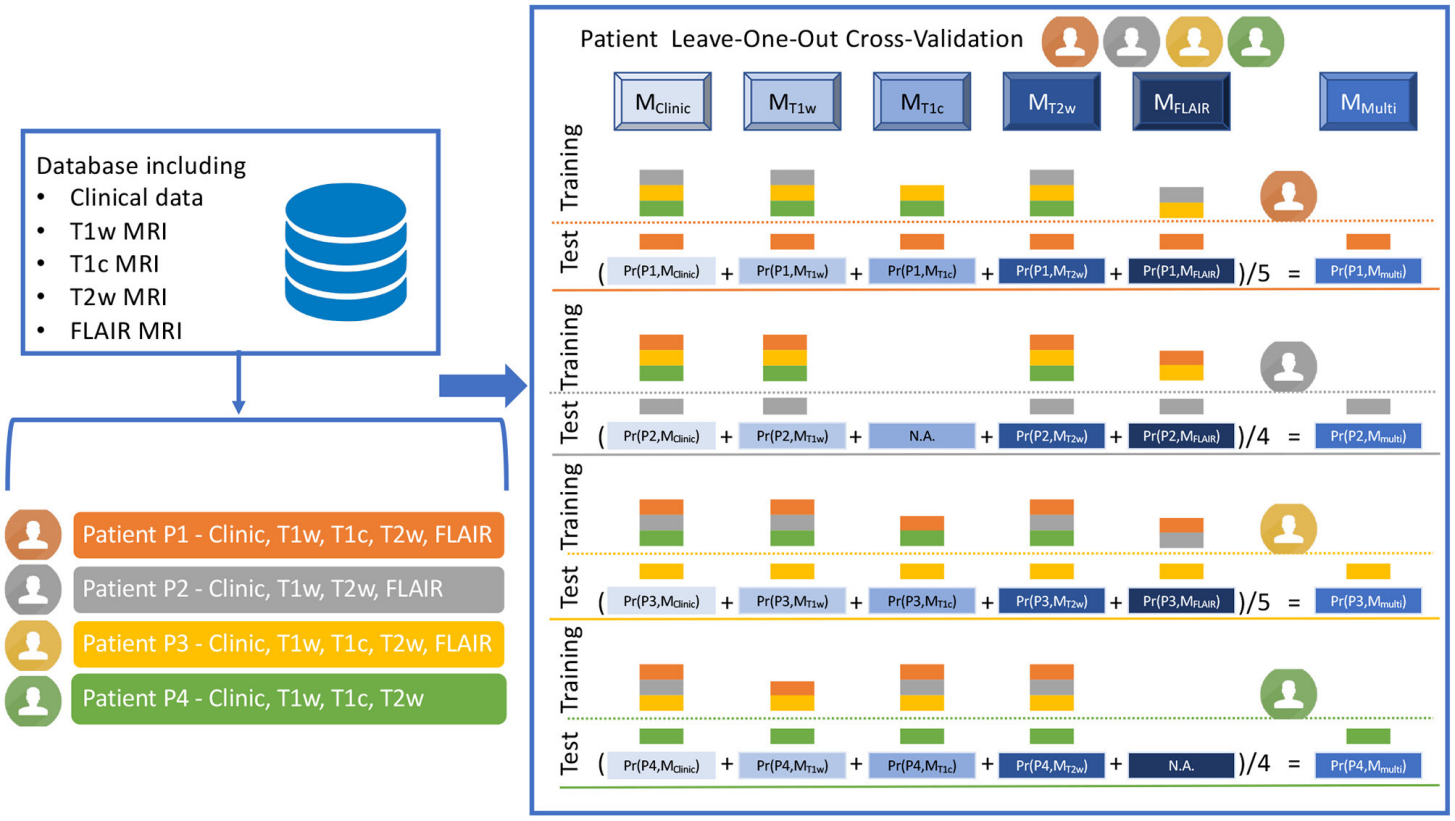
Illustration of the LOO-CV-MIM framework, i.e. the Leave-One-Out Cross-Validation framework dealing with Missing Imaging Modalities. The LOO-CV-MIM framework is applied to a binary classification task (the prediction of a mutation in our current study). The database given as a fictitious example includes four patients (P1, P2, P3, and P4, displayed in orange, gray, yellow, and green colors), P1 and P3 having the five modalities (clinical data, T1w, T1c, T2w and FLAIR MRI), P2 having one missing modality (T1c), and P4 having also one missing modality (FLAIR). For P1 (resp. P3), five models *M*_*j*_ (with *j* ∈ {*Clinic, T*1*w, T*1*c, T*2*w, FLAIR*}), are defined using as training database all the patients except P1 (resp. P3) for which the modality is present (the training database includes three patients for *M*_*Clinic*_, *M*_*T*1*w*_, and *M*_*T*2*w*_, and two patients for *M*_*T*1*c*_ and *M*_*FLAIR*_). These five models are then tested onto the remaining patient P1 (resp. P3), providing five probabilities of mutation *Pr*(*P*1, *M*_*j*_), with 0 ≤ *Pr*(*P*1, *M*_*j*_) ≤ 1 (resp. *Pr*(*P*3, *M*_*j*_)) and thus five predictions of mutation. A sixth prediction of mutation corresponding to *M*_*Multi*_, is defined as the mean value of the five probabilities *Pr*(*P*1, *M*_*j*_) (resp. *Pr*(*P*3, *M*_*j*_)). For patients P2 (resp. P4) having one missing modality, a similar process is applied but only four (and not five) models *M*_*j*_ are defined (there is no model *M*_*T*1*c*_ for P2, no model *M*_*FLAIR*_ for P4), providing four probabilities *Pr*(*P*2, *M*_*j*_) (resp. *Pr*(*P*4, *M*_*j*_)). A fifth prediction of mutation corresponding to *M*_*Multi*_, is then defined as the mean value of the four probabilities *Pr*(*P*2, *M*_*j*_) (resp. *Pr*(*P*4, *M*_*j*_)).

Due to the small number of patients and due to missing imaging modalities, a leave-one-out cross-validation (LOO-CV) framework, named LOO-CV-MIM, was proposed to compare the different models, as illustrated in Figure 3. For each training set, a logistic regression model was defined, using a L1 penalty with *C* = 0.5 (the selected features using the previously described RFE-CV procedure were frequently selected using this *C* value), and a balanced mode to automatically adjust weights inversely proportional to class frequencies of the input data. This process was applied separately to each prediction task.

To explain the process more deeply, we have to consider every patient *P*_*i*_, having *m*_*i*_ modalities such as 2 ≤ *m*_*i*_ ≤ 5, since each patient has one clinical modality and at least one among four MR modalities. For each patient *P*_*i*_, having the modality *m*, a logistic regression model $M_{m}^{i}$ is built from the *K* features $f_{m}^{k}$ selected at the previous step, the feature values inserted in the training set being computed for all the patients for which the modality *m* is available, except for the patient *P*_*i*_. The logistic regression model $M_{m}^{i}$ is then tested on the patient *P*_*i*_, providing a probability $Pr\left(P_{i},M_{m}^{i}\right)$ that the patient *P*_*i*_ had the mutation under study, according to the model $M_{m}^{i}$. Using these $Pr\left(P_{i},M_{m}^{i}\right)$ values for all the patients, and the ground truth classification, receiver operator characteristic (ROC) curve is defined and its associated area under the curve (AUC) (35) is computed as a first figure of merit. After applying the conventional threshold of 0.5 to define the final classification: if $Pr\left(P_{i},M_{m}^{i}\right)\geq 0.5$, the patient *P*_*i*_ is classified as having the mutation under study, else as not having this mutation, confusion matrices are then built. Three additional figures of merit are then computed: sensitivity, specificity (35), and balanced accuracy (mean value of sensitivity and specificity). The number of patients for which the prediction is possible is defined as an additional figure of merit.

Finally the multi-modal model approach (*M*_*Multi*_) is defined, the probability $Pr\left(P_{i},M_{Multi}^{i}\right)$ that the patient *P*_*i*_ has the mutation under study based on this ensemble model is equal to the mean probability computed for each model $M_{m}^{i}$ (see Equation 1):


(1) $$\begin{array}{c} Pr\left(P_{i},M_{Multi}^{i}\right)=\frac{1}{m_{i}}.\overset{m_{i}}{\underset{m=1}{\sum }}Pr\left(P_{i},M_{m}^{i}\right) \end{array}$$

Since the number *m*_*i*_ of models for one patient *P*_*i*_ is between 2 and 5, the $Pr\left(P_{i},M_{Multi}^{i}\right)$ term can be defined for each patient. The five figures of merit (AUC, sensitivity, specificity, balanced accuracy and number of patients for which the prediction can be done) are defined for the multi-modal model *M*_*Multi*_, too.

## 3. Results

### 3.1. Feature selection

Two clinical features (age and sex) and 79 radiomic features per imaging modality were initially considered. The RFE-CV procedure was applied to each modality (Clinical, T1w, T1c, T2w, and FLAIR) independently for the three classification tasks (prediction of H3.1, ACVR1, and TP53 mutations). From 1 to 4 features were selected per modality and resulting features are listed for each task in Tables 3–5. From clinical data, age was selected for the three tasks. For imaging modalities, in most cases, both first-order (between 1 and 2) and texture features were jointly selected. The four feature sets selected for the four MRI modalities showed some overlap across the three tasks, but none of these subsets totally overlapped. Supplementary Figure 1 displays the correlogram between the radiomic features (79 per modality) across the 61 patients selected for prediction of TP53 mutation, showing the potential interest of the four modalities, due to low or moderate correlation between features extracted from two different modalities. Supplementary Table 1 provides the features selected when merging clinical and shape features for each of the three classification tasks.

**Table 3:** Subsets of features selected by the five different models *M*_*Clinic*_, *M*_*T*1*w*_, *M*_*T*1*c*_, *M*_*T*2*w*_, and *M*_*FLAIR*_ to predict H3.1 mutation.

**H3.1 mutation**	**Features name**	**Features identifier**
*M*_*Clinic*_	Age	h1 (a1, t1)
*M*_*T*1*w*_	glcm_ClusterShade	h2
	glrlm_GrayLevelNonUniformity	h3 (a5)
	firstorder_90Percentile	h4 (a2, t4)
	firstorder_Skewness	h5 (a4)
*M*_*T*1*c*_	glcm_ClusterShade	h6 (a6)
	glrlm_ShortRunLowGrayLevelEmphasis	h7
	glszm_IntensityVariability	h8
	firstorder_10Percentile	h9
*M*_*T*2*w*_	glszm_LowIntensitySmallAreaEmphasis	h10
	glszm_HighIntensityLargeAreaEmphasis	h11 (a10)
	firstorder_TotalEnergy	h12 (a12, t9)
	firstorder_Minimum	h13
*M*_*FLAIR*_	glrlm_RunLengthNonUniformity	h14 (a15)
	firstorder_Skewness	h15 (a13)
	glcm_ClusterShade	h16
	glcm_DifferenceVariance	h17

Inside brackets, features selected by one or two other tasks of mutation prediction.

**Table 4:** Subsets of features selected by the five different models *M*_*Clinic*_, *M*_*T*1*w*_, *M*_*T*1*c*_, *M*_*T*2*w*_, and *M*_*FLAIR*_ to predict ACVR1 mutation.

**ACVR1 mutation**	**Features name**	**Features identifier**
*M*_*Clinic*_	Age	a1 (h1, t1)
*M*_*T*1*w*_	firstorder_90Percentile	a2 (h4, t4)
	glcm_Correlation	a3
	firstorder_Skewness	a4 (h5)
	glrlm_GrayLevelNonUniformity	a5 (h3)
*M*_*T*1*c*_	glcm_ClusterShade	a6 (h6)
	glszm_IntensityVariabilityNormalized	a7
	glcm_ClusterProminence	a8
	glrlm_LongRunHighGrayLevelEmphasis	a9
*M*_*T*2*w*_	glszm_HighIntensityLargeAreaEmphasis	a10 (h11)
	firstorder_Skewness	a11 (t8)
	firstorder_TotalEnergy	a12 (h12, t9)
*M*_*FLAIR*_	firstorder_Skewness	a13 (h15)
	glcm_Idmn	a14
	glrlm_RunLengthNonUniformity	a15 (h14)
	glszm_LowIntensityLargeAreaEmphasis	a16

Inside brackets, features selected by one or two other tasks of mutation prediction.

**Table 5:** Subsets of features selected by the five different models *M*_*Clinic*_, *M*_*T*1*w*_, *M*_*T*1*c*_, *M*_*T*2*w*_, and *M*_*FLAIR*_ to predict TP53 mutation.

**TP53 mutation**	**Features name**	**Features identifier**
*M*_*Clinic*_	Age	t1 (h1, a1)
*M*_*T*1*w*_	glrlm_RunLengthNonUniformity	t2
	glcm_SumAverage	t3
	firstorder_90Percentile	t4 (h4, a2)
	glszm_ZoneEntropy	t5
*M*_*T*1*c*_	glszm_HighIntensityLargeAreaEmphasis	t6
*M*_*T*2*w*_	glcm_SumAverage	t7
	firstorder_Skewness	t8 (a11)
	firstorder_TotalEnergy	t9 (h12, a12)
	glcm_AverageIntensity	t10
*M*_*FLAIR*_	glszm_IntensityVariability	t11

Inside brackets, features selected by one or two other tasks of mutation prediction.

**Figure 4:**
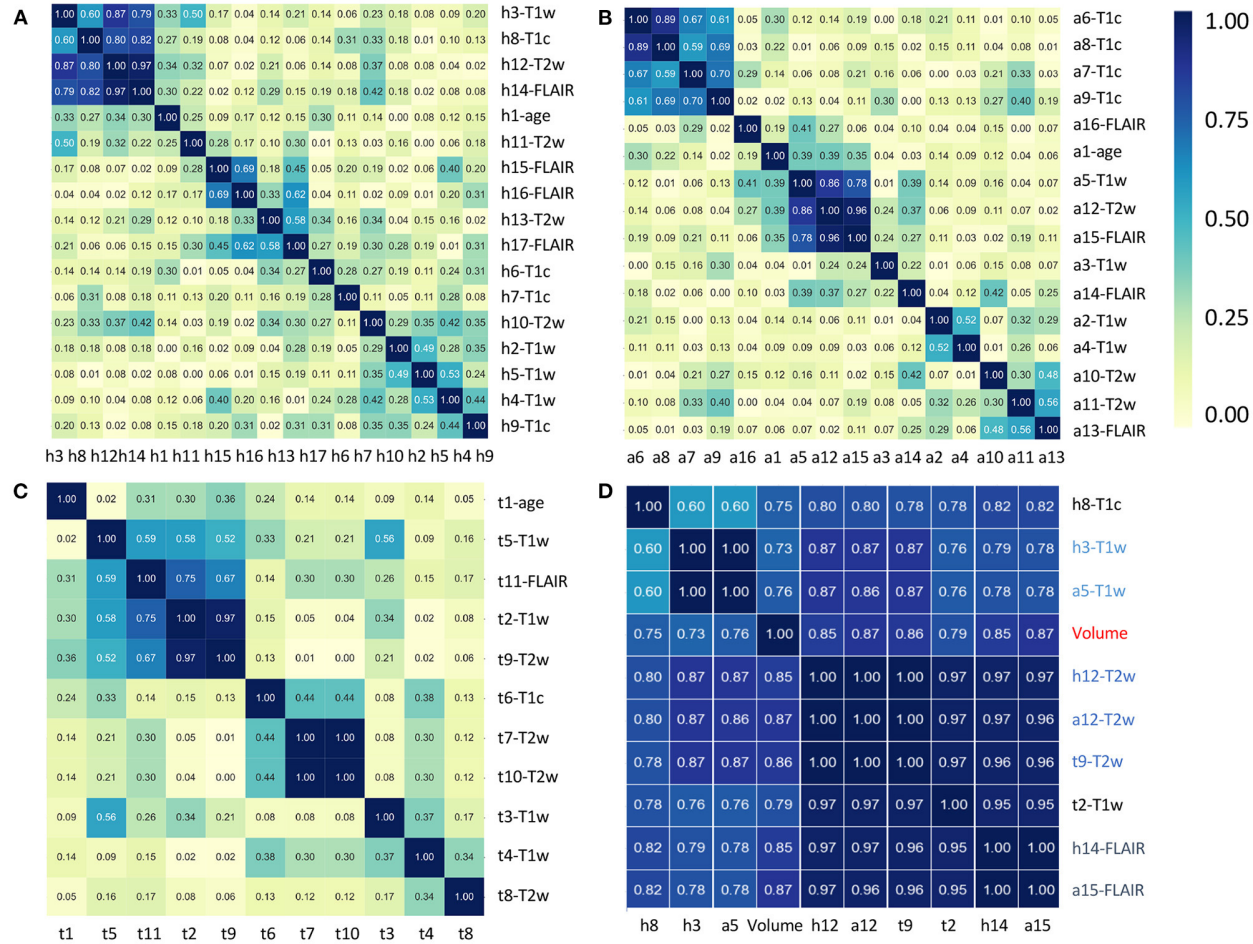
Correlation heatmaps between the features that have been selected by the five different models to predict H3.1 mutation **(A)**, ACVR1 mutation **(B)**, and TP53 mutation **(C)**. Tables 3–5 provide correspondence between feature identifiers and the full feature name according to PyRadiomics nomenclature. In **(D)**, correlation matrix heatmap between the previously selected features which are highly correlated with tumor volume. Feature identifiers (on the right side) of identical features found by the different predictive tasks are shown in color.

To further investigate the interest of each MR modality, the correlograms between the selected features are displayed in Figure 4. For the prediction of H3.1 mutation (Figure 4A), four features (h3, h8, h12, and h14) extracted from T1w, T1c, T2w, and FLAIR MRI showed high correlation. For the prediction of ACVR1 mutation (Figure 4B), three features (a5, a12, and a15) extracted from T1w, T2w, and FLAIR MRI were also highly correlated. For the prediction of TP53 mutation (Figure 4C), two features (t2 and t9) extracted from T1w and T2w MRI were also highly correlated. Interestingly, as shown in Figure 4D, all these nine features had correlation greater than 0.73 with the sphere volume, which could be considered as a surrogate marker of the tumor volume. Except for these nine features, there were no high redundancies between selected features extracted from different modalities, showing the high complementarity between these four MRI modalities. Furthermore, no selected radiomic feature was correlated with age.

### 3.2. Prediction performance

Table 6 reports the five figures of merit (number of cases, AUC, sensitivity, specificity, and balanced accuracy) obtained by the six models, for the three prediction tasks, using the LOO-CV framework. Supplementary Figures 2–4 illustrates for each patient the results of the prediction of H3.1, ACVR1, and TP53 mutations by the six types of models: *M*_*Clinic*_, *M*_*T*1*w*_, *M*_*T*1*c*_, *M*_*T*2*w*_, *M*_*FLAIR*_, and *M*_*Multi*_. Supplementary Table 2 displays the five figures of merit for two additional models: *M*_*ClinicSh*_ and *M*_*MultiSh*_ for which the shape features were merged with the clinical features.

**Table 6:** Prediction results for the six models: *M*_*Clinic*_, *M*_*T*1*w*_, *M*_*T*1*c*_, *M*_*T*2*w*_, *M*_*FLAIR*_, and *M*_*Multi*_ in a LOO-CV framework.

**Models**	***M*_*Clinic*_**	***M*_*T*1*w*_**	***M*_*T*1*c*_**	***M*_*T*2*w*_**	***M*_*FLAIR*_**	***M*_*Multi*_**
**H3.1 mutation**						
Number of patients	**63** (14)	58 (13)	58 (14)	59 (13)	56 (12)	**63** (14)
AUC	0.82	0.87	**0.92**	0.71	0.74	0.91
Sensitivity (%)	85.7	84.6	78.6	92.3	83.3	**100**
Specificity (%)	57.1	**75.6**	77.3	52.2	72.7	75.5
Balanced Accuracy (%)	71.4	80.1	77.9	72.2	78.0	**87.8**
**ACVR1 mutation**						
Number of patients	**63** (14)	58 (13)	58 (13)	59 (13)	56 (13)	**63** (14)
AUC	0.74	0.78	0.77	0.72	0.83	**0.91**
Sensitivity (%)	85.7	76.9	76.9	84.6	76.9	**92.9**
Specificity (%)	44.9	60.0	71.1	58.7	**74.4**	71.4
Balanced Accuracy (%)	65.3	68.5	74.0	71.7	75.7	**82.1**
**TP53 mutation**						
Number of patients	**61** (34)	56 (33)	57 (32)	57 (33)	54 (32)	**61** (34)
AUC	0.78	0.83	0.63	0.86	0.75	**0.88**
Sensitivity (%)	55.9	66.7	28.1	69.7	**71.9**	67.6
Specificity (%)	85.2	78.3	88.0	87.5	54.5	**88.9**
Balanced Accuracy (%)	70.5	72.5	58.1	**78.6**	63.2	78.3

For each prediction task and for each model, five figures of merit are reported: the total number of patients for which the prediction was possible (the number of patients with mutation is between brackets), the AUC under the ROC curve, the sensitivity, the specificity and the balanced accuracy. For each figure of merit, best results are in bold characters and second best results are underlined.

Three points emerge from the analysis of these results.

#### 3.2.1. Radiomics increase the performance of the predictors

Indeed, the simple clinical feature “age” provided alone some pretty good results with a balanced accuracy equal to 71.4% for predicting H3.1 mutation, 70.5% for predicting TP53 mutation and 65.3% for predicting ACVR1 mutation. These values could be considered as baseline. When compared to baseline, adding MR radiomic data through the multi-modal model enabled an increase of 16 percentage points of the balanced accuracy for predicting H3.1 and ACVR1 mutations and of 8 percentage points for predicting TP53 mutation. Finally, the addition of the shape radiomic features slightly improved the prediction of TP53 mutation, with an increase of 1.4 percentage point of the balanced accuracy.

#### 3.2.2. Ensembled multi-modal model outperforms mono-modal predictors

Noticeably the multi-modal approach provided the best (or second best) performances for all the figures of merit whatever the predictive tasks. Thanks to its inception, it provided a prediction for each patient, even in case of missing MR data. Following results in Table 6, missing MR data varies between 6 and 11%, according to the MR modality and the task of prediction. The AUC associated with the *M*_*Multi*_ model was the highest for predicting ACVR1 (0.91) and TP53 (0.88) mutations, and the second highest for predicting H3.1 mutation (0.91 vs. 0.92 for *M*_*T*1*c*_). Sensitivity was the highest for predicting H3.1 and ACVR1 mutation. It reaches the third position for predicting TP53 mutation (67.6 vs. 69.7% for *M*_*T*2*w*_ and 71.9% for *M*_*FLAIR*_), but for that task, it achieves the highest specificity. Taking into account the balanced accuracy as a compromise between sensitivity and specificity, this figure of merit was the highest for predicting H3.1 mutation (87.6%) and ACVR1 mutation (82.1%) and the second highest for predicting TP53 mutation (78.3 vs. 78.6% for *M*_*T*2*w*_), having a prediction for the 61 patients vs. 57 for *M*_*T*2*w*_. The same effects were observed when the clinical features were replaced by the clinical and the shape features, showing the value of the multi-modal model in a slightly different configuration.

#### 3.2.3. Each MR modality brings specific information

Depending on the task, the ranking of the four models built from each MR modality varied. For instance, the T2w modality appears to be less relevant for predicting H3.1 and ACVR1 mutations, but it proves to have very high figures of merit for the prediction of TP53 mutation. The FLAIR modality appears to be very relevant for predicting ACVR1 mutation but less relevant for predicting TP53 mutation. Furthermore, the shape features which could be extracted without missing values could have an impact for predicting TP53 mutation, too. These results underline the necessity to acquire all the structural modalities to achieve multi-objective classification tasks.

## 4. Discussion

The proposed approach provides a good prediction of three important mutations (H3, ACVR1, and TP53) encountered in patients with DIPG, within a constrained experimental setting including missing data and small cohort. This result could have a real impact in the coming years to propose a more personalized therapy to patients with DIPG. Our approach is based on clinical and MR data and could be applied in case of absent or not conclusive biopsy. As reported in the literature (1), age was shown to be a relevant predictor of the three mutations, but this study shows that some radiomic models can outperform this baseline predictor, with radiomics originated from T1w, T1c, and FLAIR for H3.1 mutation, T1c and FLAIR for ACVR1 mutation, and T2w and the shape features for TP53 mutation (see Table 6 and Supplementary Table 2). With our ensembled multi-modal approach, a prediction can be done for each patient, even if she/he lacks one or more MR modalities, and all the figures of merit were among the highest. In the LOO-CV framework, the number of false positive and false negative cases was reduced to 19% (resp. 24 and 23%) for the prediction of H3.1 (resp. ACVR1 and TP53) mutations. This DIPG study illustrates thus the positive impact of radiomic approaches for these three predictive tasks.

From a methodological point of view, radiomic studies rely on a succession of steps which have to be optimized. As our database is small, several methods are admissible and can bring some equivalent solutions. Users are recommended to follow best practices (36), some of which depending on MRI. In clinical studies involving MRI, we have demonstrated the interest of MR data preprocessing with image standardization (21, 37) and radiomic feature harmonization (26, 28) to provide more comparable features across scanners, sequences and patients. Furthermore, if automatic tumor segmentation is a major issue to solve and requires additional developments, the precision of segmentation that is required depends on the task to solve. It appears for this study of mutation prediction in DIPG, the definition of a large sphere inside the tumor was sufficient to provide good results and the fine delineation of the tumor in 3D was not absolutely necessary for this discovery step. For feature selection, several approaches are possible. Using a different approach based on feature filtering (and not on RFE-CV) in some preliminary works (29, 32), we found that similar features were found to be predictive of H3.1 mutation. As there are many correlated features for the same MR modality (as shown in Supplementary Figure 1), some equivalent models can be defined using different sets of features.

This study shows also a pragmatic but efficient approach to deal with missing (or insufficient quality data) MR modalities, while taking advantage of the complementarity among them. Our objective was to use all the information that was available without data rejection or data imputation. Data rejection, for instance removing patients having <4 MR modalities, would have considerably reduced the number of cases (from 80 to 57 patients), and therefore likely decreased the performance of the models (38). In their recent study related to prediction of H3K27M mutation in diffuse midline glioma using multi-modal MRI, more than 50% of patients were excluded due to missing data or insufficient quality (39). Our multi-modal model could remedy such a situation, and enable studies with larger number of patients providing more robust results. Among other conventional approaches used to deal with missing data, MR data imputation appeared to be complex for two main reasons: the low number of cases that were initially available, and the low correlation between the features coming from different modalities (except from those which are highly correlated with the volume or the shape of the region), as underlined by Figure 4. For similar reasons, generative adversarial networks (40) to synthesize missing MR volumes were not retained as a feasible option.

In our preliminary work (32), 16 models were defined to deal with missing data for the prediction of H3.1 mutation: one clinical model based on age, four mono-modal models combined with age and 11 additional models merging two (providing 6 models), three (providing 4 models) and four (providing 1 model) MRI modalities. However, these 11 additional models proved to be redundant with the 4 mono-modal models since they were based on very similar sets of features. Thus, the majority voting on all possible models that was applied to each patient could be partially biased.

Radiogenomic studies in neuro-oncological studies (41) have shown a small number of studies devoted to DIPG or diffuse midline glioma (DMG). For the specific classification tasks we aimed at solving, we did not find any strictly comparable studies. Indeed, if several studies (39, 42–44) have proposed some radiomic models to distinguish between H3K7M mutation and Histone H3 Wild-Type groups, all of them included an adult population with DMG, which manifest themselves in several different ways compared to pediatric cancers. Therefore, features and models proposed by those studies could not be compared with ours. Furthermore, we did not find any study aiming at predicting ACVR1 mutations or TP53 mutations in patients with DIPG or DMG.

Our study presents several limitations. Despite the selection of a reduced number of features (4 or less features per mono-modal model), some over-fitting could still be present, especially for the prediction of H3.1 and ACVR1 mutations, for which the data sets were strongly imbalanced. However, we are confident in the interest of the multi-modal model, since it proves its superiority for the three different tasks considered here. As the different mono-modal models have the same weight in the definition of the multi-modal approach, optimizing their weight according to their performances could also be tested. However, following this direction, first attempts consisting in removing the 'worst' modality did not show any significant changes. The radiological interpretation of selected features, apart those close to volume or shape, needs also to be refined. For this point, we should test the use of decision maps, as recently introduced in (45). Furthermore, a recent study (46) has shown the superiority of segmenting tumor volume over its ellipsoidal approximation to assess tumor burden in DIPG. The fine delineation of contours will make possible to further test the impact of additional morphological features, including the histogram of oriented gradients as proposed by Alksas et al. (47) for the estimation of the genomic mutations. The manual segmentation is however tedious and its reproducibility still needs to be tested. This task is also difficult to automate due to the particularities of DIPG and the difficulties of obtaining a cohort with numerous data (48). Finally, several works remain to be done. To get rid of the data leakage which was present in our feature selection, the external test of our different models should be done to validate them or to propose some simplified models to travel across the different centers. The model of logistic regression was chosen due to its simplicity and its robustness, and this choice proves to be informative in our context of small number of cases and of imbalanced classes. Regarding prediction performance, our results are certainly overestimated, especially with the LOO-CV process. With a larger database, the performances will be better assessed, and different machine learning models could also be tested, tuned, and compared. Measuring the added value of perfusion and diffusion studies (49), for which the number of missing modalities will be higher, is also a challenge to solve. The interest of MR radiomics to define prognosis (50, 51) should also be further analyzed when compared to simpler models (50, 52, 53).

## 5. Conclusion

The interest of using MRI radiomics in addition to clinical data to predict mutations of H3.1, ACVR1 and TP53 was shown on a retrospective cohort of 80 subjects. Each MR modality (T1w, T1c, T2w, and FLAIR) demonstrates its interest for at least one of the three prediction tasks. Compared to single-modal models, the multi-modal model combining multiple MRI modalities and clinical features was the most powerful and could provide a prediction for every patient, even in the case of missing MR modalities. It could thus be tested as an alternative in the absence of biopsy or in case of non-conclusive results of the genetic analysis.

## Data availability statement

The original contributions presented in the study are included in the article/Supplementary material, further inquiries can be directed to the corresponding author.

## Ethics statement

The studies involving human participants were reviewed for the BIOMEDE clinical trial (NCT02233049), which includes neuroimaging. It was approved by a French Ethics Committee: Comité de Protection des Personnes (CPP). The CPP of Ile de France III provided an approval on 25 August 2014 (approval ID #2014-001929-32). Written informed consent to participate in this study was provided by the participants' legal guardian/next of kin.

## Author contributions

FF and FK wrote the manuscript draft. FF and VF designed the study. VD-R and NB provided image data and their radiological expertise. JG provided molecular data and his medical expertise. AG, CP, and VF built the image database. FK, JG-O, and TE proposed and implemented MR data processing. All authors approved it.
