# Supplementary material for: Multimodal MRI radiomic models to predict genomic mutations in diffuse intrinsic pontine glioma with missing imaging modalities
Source: Front Med (Lausanne). 2023 Feb 23;10:1071447. doi: 10.3389/fmed.2023.1071447 (PMC9995801; doi:10.3389/fmed.2023.1071447)
Supplement: Supplementary file 1 [file Data_Sheet_1.PDF]

Supplementary Material

SUPPLEMENTARY DATA

Supplemental file DIPGFeatures.xlsx contains the clinical and biological information, and the radiomic features used for the present study. The first sheet, entitled Classif, provides for each of the 80 subjects, subject code, MR field of the scanner, sex (0 for female, 1 for male), age (in years), volume of tumor (in mm^3), H3 mutation (1 for H3.1, 2 for H3.2, 3 for H3.3, 0 for H3 Wild-Type, na when unknown), ACVR1 mutation (1 for ACVR1 mutation, 0 for Wild-Type, na when unknown), TP53 mutation (1 for TP53 mutation, 0 for Wild-Type, na when unknown). Then T1w, T1c, T2w, and FLAIR entitled sheets provide the 79 radiomic features after extraction from T1w, T1c, T2w and FLAIR images (without ComBat transformation), for each subject having the corresponding modality.

SUPPLEMENTARY TABLES AND FIGURES

**Classification task**	Features name	Features identifier
H3.1 mutation	Age	h1 (a1, t1)
	shape_Elongation	h18 (a17)
	shape_Flatness	h19 (t12)
	shape_SurfaceVolumeRatio	h20 (a19, t13)
ACVR1 mutation	Age	a1 (h1, t1)
	shape_Elongation	a17 (h18)
	shape_Maximum2DDiameterRow	a18
	shape_SurfaceVolumeRatio	a19 (h20)
TP53 mutation	Age	t1 (h1, a1)
	shape_Flatness	t12 (h19)
	shape_SurfaceVolumeRatio	t13 (h20)

Table S1. Subsets of features selected by the model $M_{ClinicSh}$ merging clinical features and shape features to predict H3.1, ACVR1, and TP53 mutations. Inside brackets, features selected by one or two other tasks of mutation prediction.

Models	M_{Clinic}	$M_{ClinicSh}$	M_{Multi}	$M_{MultiSh}$
**H3.1 mutation**				
Number of patients	63 (14)	63 (14)	63 (14)	63 (14)
AUC	0.82	0.87	0.91	**0.95**
Sensitivity (%)	85.7	92.9	**100**	**100**
Specificity (%)	57.1	61.2	**75.5**	73.5
Balanced Accuracy (%)	71.4	77.0	**87.8**	86.7
**ACVR1 mutation**				
Number of patients	63 (14)	63 (14)	63 (14)	63 (14)
AUC	0.74	0.80	0.91	**0.93**
Sensitivity (%)	85.7	**92.9**	**92.9**	**92.9**
Specificity (%)	44.9	55.1	**71.4**	67.3
Balanced Accuracy (%)	65.3	74.0	**82.1**	80.1
**TP53 mutation**				
Number of patients	61 (34)	61 (34)	61 (34)	61 (34)
AUC	0.78	0.83	0.88	**0.91**
Sensitivity (%)	55.9	64.7	67.6	**70.6**
Specificity (%)	85.2	**88.9**	**88.9**	**88.9**
Balanced Accuracy (%)	70.5	76.8	78.3	**79.7**

Table S2. Prediction results for four models: M_{Clinic}, as defined in the main text, which is based on age only, $M_{ClinicSh}$, combining clinical features and 14 shape features, M_{Multi} as defined in the main text, including clinic and intensity and texture radiomic features, $M_{MultiSh}$ including clinic, shape, intensity and texture radiomic features, in the LOO-CV-MIM framework. For each prediction task and for each model, the five figures of merit defined in the main text are reported: the number of patients (all of them for the four models), the AUC of the ROC curve, the sensitivity, the specificity and the balanced accuracy. The best performances (in boldface) are provided by the multi-model approaches. There is no clear improvement brought by the introduction of the shape features in the prediction of the H3.1 and ACVR1 mutations. However, there is a slight trend of improvement for the prediction of the TP53 mutation.

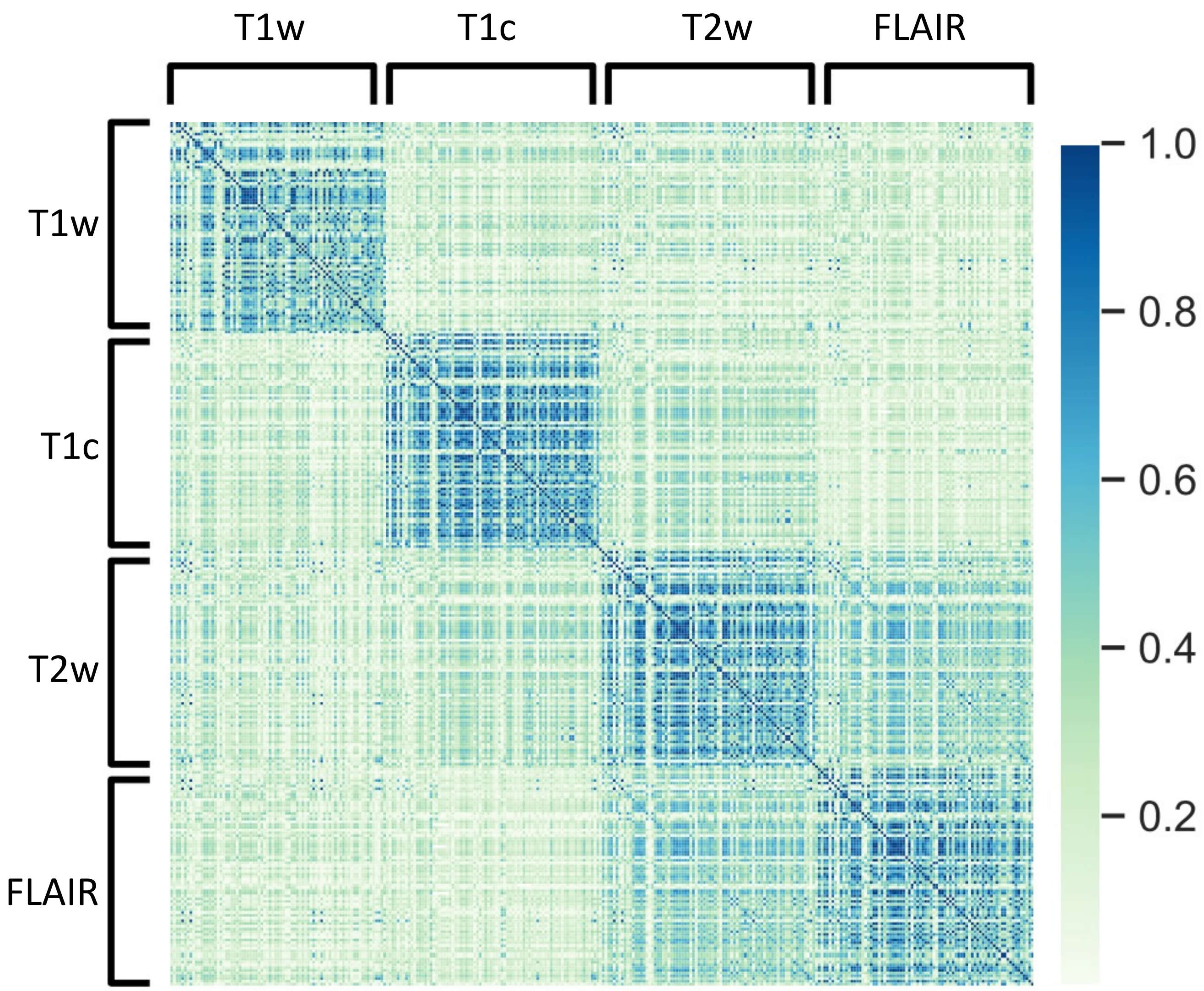

Figure S1. Correlation matrix heatmap between the 4x79 features computed for each modality: T1w, T1c, T2w, and FLAIR. The general trend is that the correlations between two features from two different modalities is less than the correlation between two features from the same modalities and when comparing modalities two by two, the features computed for T2w and FLAIR modalities seem to be the most correlated.

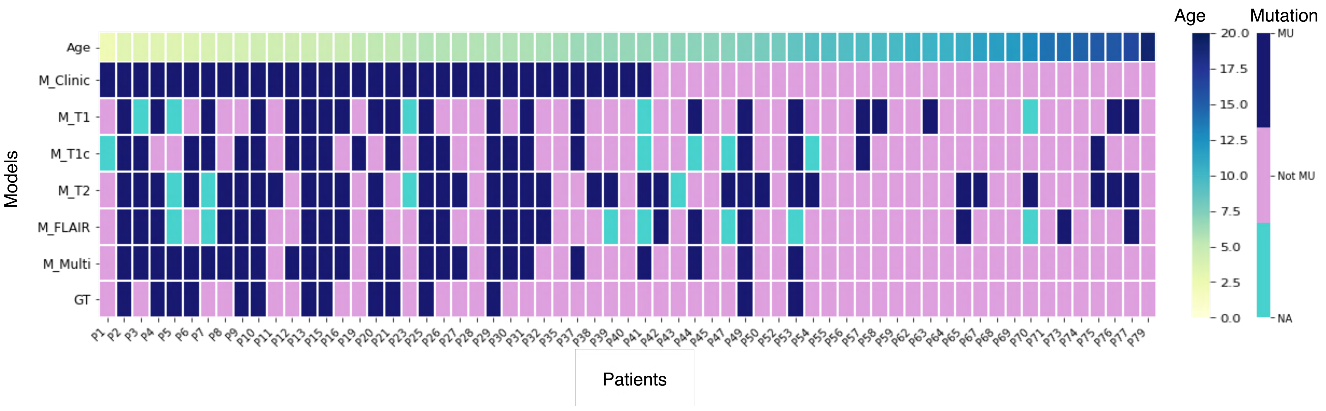

Figure S2. Predictive results of H3.1 mutation by the six models for each patient of the database having a ground truth (n=63). Patients (one per column, being ordered by age) along with the age distributions (yellow/green/blue color). From top raw to bottom raw: age, binary output of the six predictive models M_{Clinic}, M_{T1w}, M_{T1c}, M_{T2w},M_{FLAIR}, M_{Multi} and ground truth (GT). MU (dark blue color) stands for mutated cases, Not MU (pink color) stands for not H3.1 mutated cases, NA (cyan color) stands for not available modality.

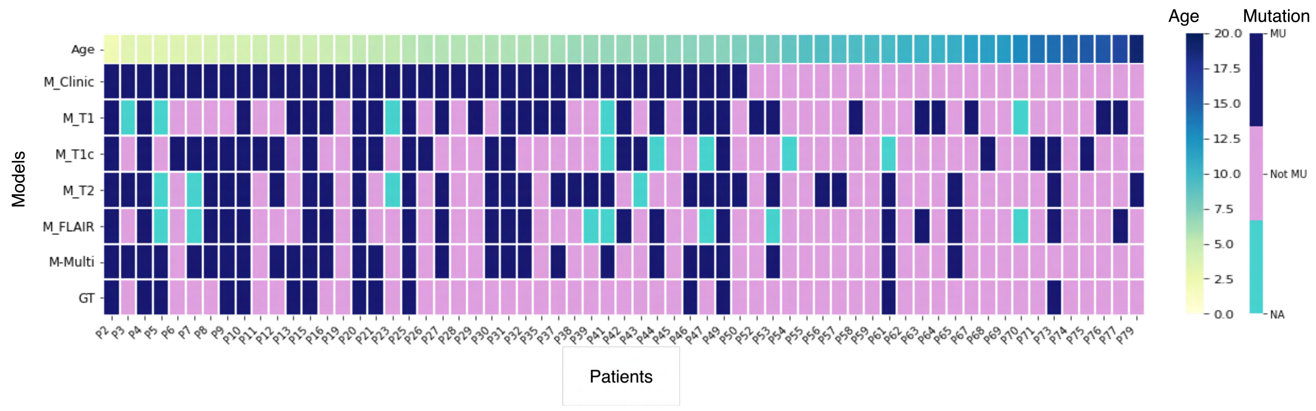

Figure S3. Predictive results of ACVR1 mutation by the six models for each patient of the database, having a ground truth (n=63). Patients (one per column, being ordered by age) along with the age distributions (yellow/green/blue color). From top raw to bottom raw: age, binary output of the six predictive models M_{Clinic}, M_{T1w}, M_{T1c}, M_{T2w},M_{FLAIR}, M_{Multi}, and ground truth (GT). MU (dark blue color) stands for mutated cases, Not MU (pink color) stands for not mutated cases, NA (cyan color) stands for not available modality.

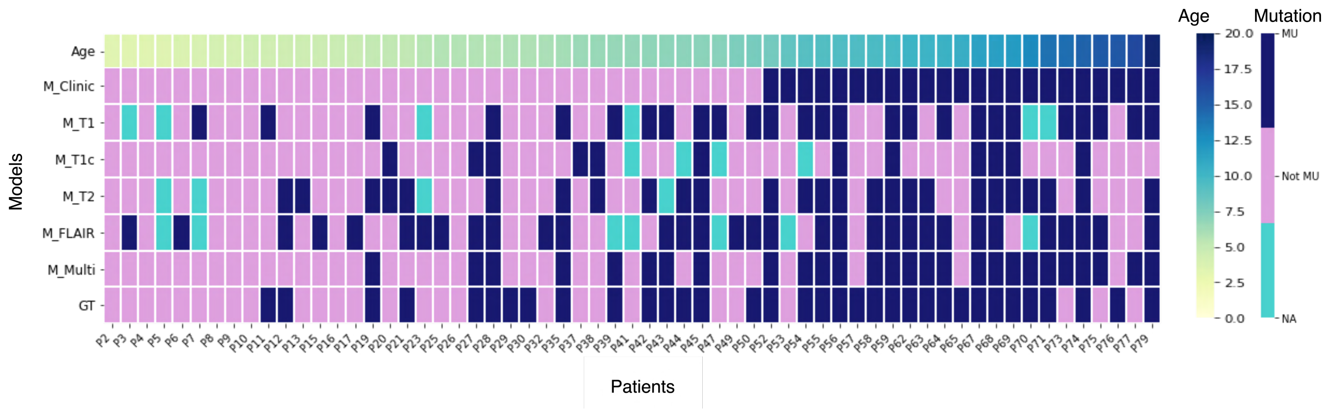

Figure S4. Predictive results of TP53 mutation by the six models for each patient of the database, having a ground truth (n=61). Patients (one per column, being ordered by age) along with the age distributions (yellow/green/blue color). From top raw to bottom raw: age, binary output of the six predictive models M_{Clinic}, M_{T1w}, M_{T1c}, M_{T2w},M_{FLAIR}, M_{Multi} and ground truth (GT). MU (dark blue color) stands for mutated cases, Not MU (pink color) stands for not mutated cases, NA (cyan color) stands for not available modality.
